# Glucose transport by epithelia prepared from harvested enterocytes

**DOI:** 10.1007/s10616-013-9656-1

**Published:** 2013-10-29

**Authors:** Yasuhiro Kimura, Marie van der Merwe, Stine B. Bering, Himabindu Penmatsa, Veronica G. Conoley, Per T. Sangild, Anjaparavanda P. Naren, Randal K. Buddington

**Affiliations:** 1 Department of Food and Nutrition, Beppu University, 82 Kita-Ishigaki, Beppu, Oita 874-8501 Japan; 2 Department of Health and Sport Sciences, University of Memphis, 306 Elma Roane Fieldhouse, 495 Zach Curlin Wy, Memphis, TN 38152-3480 USA; 3 Department of Nutrition, Exercise and Sports, University of Copenhagen, Frederiksberg C, Denmark; 4 Department of Physiology, University of Tennessee Health Science Center, Memphis, TN 38163 USA

**Keywords:** Cell culture, Intestine, Enterocyte, Nutrient transport

## Abstract

Transformed and cultured cell lines have significant shortcomings for investigating the characteristics and responses of native villus enterocytes in situ. Interpretations of results from intact tissues are complicated by the presence of underlying tissues and the crypt compartment. We describe a simple, novel, and reproducible method for preparing functional epithelia using differentiated enterocytes harvested from the small intestine upper villus of adult mice and preterm pigs with and without necrotizing enterocolitis. Concentrative, rheogenic glucose uptake was used as an indicator of epithelial function and was demonstrated by cellular accumulation of tracer ^14^C d-glucose and Ussing chamber based short-circuit currents. Assessment of the epithelia by light and immunofluorescent microscopy revealed the harvested enterocytes remain differentiated and establish cell–cell connections to form polarized epithelia with distinct apical and basolateral domains. As with intact tissues, the epithelia exhibit glucose induced short-circuit currents that are increased by exposure to adenosine and adenosine 5′-monophosphate (AMP) and decreased by phloridzin to inhibit the apical glucose transporter SGLT-1. Similarly, accumulation of ^14^C d-glucose by the epithelia was inhibited by phloridzin, but not phloretin, and was stimulated by pre-exposure to AMP and adenosine, apparently by a microtubule-based mechanism that is disrupted by nocodazole, with the magnitudes of responses to adenosine, forskolin, and health status exceeding those we have measured using intact tissues. Our findings indicate that epithelia prepared from harvested enterocytes provide an alternative approach for comparative studies of the characteristics of nutrient transport by the upper villus epithelium and the responses to different conditions and stimuli.

## Introduction

The epithelium lining the intestine is a critical interface between the luminal environment and the host that adapts to changes in functional demands during the life history of individuals and evolution of species. The epithelium serves multiple roles, including digestion, osmoregulation, immune and endocrine functions, and detoxification. Moreover, many intestinal diseases can be tracked to a dysfunction of the villus epithelium. Yet, our understanding of the absorptive and other functions of the intestinal epithelium is limited to either in vivo studies, intact tissues or stripped mucosa that are complicated by the presence of multiple tissue layers and both crypt and villus compartments, or to the use of transformed human cell lines with enterocyte-like characteristics (e.g., Caco-2 and HT-29 cells) and non-transformed primary intestinal epithelial cell (IEC) lines that are derived from proliferating crypt cells of a limited number of species and usually from fetuses (Liu et al. 2010; Grossman et al. 2003).

There is a need for in vitro preparations of normal, differentiated enterocytes that are representative of the in situ villus epithelium and are suitable for comparative studies. Although transformed cell lines are used as models of enterocytes, there is justifiable concern about their relevancy to in situ characteristics and responses (Chougule et al. 2012). For example, adenosine causes a rapid, non-genomic increase in glucose transport in vivo and by intact small intestine (Kimura et al. 2005), but decreases glucose accumulation by Caco-2 cells by 70 % or more (our unpublished data), which led us to search for more relevant alternative cell models. Short (Bader et al. 2000) and long-term (Panja 2000) cultures of IEC have been prepared from stem cells harvested from fetal tissues (Kaeffer 2002), with some success using tissues from older life stages (Weng et al. 2005), including adult humans (Chougule et al. 2012). Proliferating, IEC can be induced to form epithelia, exhibit characteristics of differentiated enterocytes, and remain viable for up to 10 days, and even longer. However, the long term, aseptic culture, and need to induce differentiation of IEC is likely to result in cells adapted to culture conditions and with characteristics unlikely to reflect those of native enterocytes exposed to the complex milieu of the intestinal contents and the regulatory molecules of the host. Recently described organoids (Chopra et al. 2010) grown from isolated crypt stem cells have proven valuable, but suffer some of the same limitations as IEC. As a consequence, the characteristics of epithelia consisting of native villus enterocytes and the responses to luminal and host regulatory molecules have been elusive (Perreault and Beaulieu 1998). Moreover, because current cell lines are derived from a limited number of species, they provide only limited insights into comparative differences among species, ages, and health states.

We report herein the preparation of epithelia using enterocytes harvested from the upper villus of adult mice and from preterm pigs of different health states. Our measurements of glucose uptake demonstrate that the epithelia provide a simple, reproducible, and sensitive alternative approach for comparative studies of the nutrient transport characteristics of enterocytes and the villus epithelium, and for examining the responses to luminal stimuli, host regulatory signals, and to therapeutic and environmental molecules.

## Materials and methods

### Harvesting and culture of enterocytes

All aspects of the research using animals had been approved by the Institutional Animal Care and Use Committees where the animals were housed (University of Memphis, University of Tennessee Health Sciences Center, and University of Copenhagen). Adult mice of the C57BL/6 strain were selected because of widespread use for transgenic applications (Fielder et al. 2010). Preterm pigs (delivered at 92 % of gestation) that were either healthy or diagnosed with the gastrointestinal inflammatory disease necrotizing enterocolitis (NEC) localized to the colon were included for comparative purposes.

The approach to harvesting the enterocytes was adapted from Bader et al. (2000). The small intestine was removed immediately after euthanasia (CO_2_ asphyxiation for mice and a combination of sodium pentobarbital and phenytoin sodium (Euthasol) for pigs; 1 ml/kg) and the digesta were flushed out with ice cold Dulbecco’s Phosphate Buffered Saline (DPBS). The small intestine of mice or a segment of jejunum from preterm pigs was everted and incubated for 10 min with gentle agitation in room temperature DPBS with 0.05 % dithiothreitol. Afterwards, the segments were rinsed with room temperature DPBS, then incubated with gentle agitation in a mixture of equal proportions of an enzyme-based dissociation/cell detachment solution (HyQTase^®^; HyClone, Logan, UT, USA) and an enzyme-free solution (Enzyme free cell dissociation solution, PBS based; Millipore; Billerica, MA, USA) for 15–30 min at 37 °C to release enterocytes from the villus epithelium.

The dissociation solution with the released cells was poured into a centrifuge tube with pre-chilled DPBS through 4 layers of cheese cloth to remove clumped cells. The cells were sedimented (200×g; 3–4 min, 4 °C), the supernatant was aspirated and the cells were rinsed 3× by suspending in ice cold DPBS, sedimenting, and removing the supernatant. After the third rinse, the cells were suspended in DPBS with 0.2 % bovine serum albumin and the density of isolated cells was determined using a Scepter 2.0 cell counter (Millipore) and viability by trypan blue exclusion. The cells were sedimented a final time, the DPBS with bovine serum album was removed, and the cells were suspended at a density of 10 million enterocytes per ml in cold low glucose Dulbecco’s modified Eagle’s medium (DMEM; Gibco, Life Technologies, Grand Island, NY, USA; Hyclone, Logan, UT, USA; Lonza, Allendale, NJ, USA) with 10 % sterile filtered fetal bovine serum (FBS; Thermo Fisher Scientific, Waltham, MA, USA; Sigma Chemical Co., St. Louis, MO, USA), 1 ml of a combination of penicillin (100 U/ml) and streptomycin (100 μg/ml), 5 ml of 4-(2-hydroxyethyl)-1-piperazineethanesulfonic acid (HEPES; 10 mM; pH = 7.3), and a commercial combination of insulin, transferrin, and selenium (ITS, added per supplier instructions; HyClone or Lonza). The culture media did not contain prednisolone or other corticosteroids. DMEM was selected as the basal medium based on the widespread use for culture of mammalian cells (Kaeffer 2002), though DMEM is sometimes combined with F12 medium (Chougule et al. 2012).

The cell suspensions were added to uncoated 0.33 and 0.6 cm^2^ polycarbonate membrane inserts (Millipore) to achieve a seeding density of 4–5 million cells per cm^2^. The inserts were placed in culture plates, the lower wells were filled with the culture medium, and incubated at 37 °C in 95 % room air with 5 % CO_2_.

### Microscopic evaluation

Suspensions of cells prior to plating were examined by light microscopy to assess the proportion of solitary cells. Segments of mouse intestine before and after the harvest of enterocytes were fixed in 10 % neutral buffered formalin and processed for light microscopy to examine where the cells were removed from the villi.

The proportion of harvested cells represented by enterocytes was determined using a combination of flow cytometry (FACS) and immunocytochemistry. The percentage of cells from epithelial origin was based on presence of the epithelial cell marker EpCAM (Chougule et al. 2012). The Fc receptors were blocked using rat anti-mouse CD16/CD32 (clone 2.4G2, BD Biosciences; San Jose, CA, USA) before the cells were labeled with FITC-conjugated anti-CD326 (EpCAM, clone G8.8, Biolegend; San Diego, CA, USA) or with FITC-conjugated rat IgG2a as the isotype control (clone RTK2758, Biolegend). Goblet cells were detected by probing the harvested cells for intracellular Trefoil Factor 3 (TFF3; Hoffman et al. 2001). After EpCAM labeling, cells were fixed with fixation buffer (Biolegend) and permeabilized with a permeabilization wash buffer (Biolegend) as per the manufacturers’ instructions. The intracellular Fc receptors were blocked with rat anti-mouse CD16/CD32 (BD Biosciences) and the cells were labeled with PE-conjugated anti-TFF3 (1 μg per 1 × 10^6^ cells) (cat. number bs-0535R, Bioss Inc, Boston, MA, USA) or PE-conjugated mouse IgG1 isotype control (clone MOPC-21, Biolegend). The labeled cells were analyzed using BD LSR-II I^®^ instrument (BD Biosciences), and the data were analyzed using FlowJo^®^ software (Treestar, Eugene OR, USA). For immunocytochemistry, cells were stained as mentioned above. In addition, 4′,6′-Diamidino-2-phenylindole (DAPI, Biolegend) was added and the cells were allowed to attach to poly-l-lysine coated microscope slides (Thermo Fisher Scientific). Cells were observed using a Zeiss Axioimager M2 with an AxioCam Mrc (Carl Zeiss Microscopy, Thornwood, NY, USA) and images were analyzed using Axiovision Rel 4.8 software.

The presence of tight junctions was visualized by immunofluorescent staining for Zonula Occludens**-**1 (ZO-1). Briefly, epithelia were fixed at 4, 9, and 20 h post seeding using acetone:methanol (1:1). The cells were permeabilized with 0.2 % Triton-X 100 in PBS before exposure to a ZO-1 mouse monoclonal antibody (Zymed Laboratories, Inc., South San Francisco, CA, USA) at a dilution of 1:1,000. After rinsing, a secondary goat-anti-mouse antibody (Alexa 488; Molecular Probes Inc., Eugene, OR, USA) was applied at a 1:150 dilution. Both antibodies were diluted in 3 % fat-free milk in PBS and exposure conditions were 1 h for both antibodies in a dark humidifying chamber.

### Functional characteristics

Transepithelial electrical resistance (TEER) was measured as a function of cell density (3.5 × 10^5^–6 × 10^6^ enterocytes cm^−2^) and time after plating (0–12 h) using an EVOM^®^ Epithelial Voltohmmeter (World Precision Instruments, Sarasota, FL, USA) and quadruplicate inserts plated with cells harvested from mouse small intestine. Based on these measurements, additional assessments of the electrical properties of the epithelia and glucose-induced currents were made using a vertical Ussing chamber system (Physiologic Instruments, San Diego, CA, USA) beginning 6–8 h after culturing cells on 0.66 cm^2^ inserts at densities of 4–5 × 10^6^ enterocytes cm^−2^. The apical and basolateral chambers were filled with 37 °C mammalian Ringers aerated with a gas mixture of 95 % O_2_ and 5 % CO_2_. After a stable baseline current was observed, a portion of the apical solution was replaced with a 50 mM solution of glucose in Ringer’s (prepared by isosmotic replacement of a portion of the NaCl) to achieve a 20 mM concentration of glucose in the apical chamber. Simultaneously, a portion of the basolateral medium was similarly exchanged with a solution of 50 mM mannitol in Ringers to an identical final concentration of 20 mM. The resulting short circuit current (*Isc*) response was considered indicative of the rheogenic sodium–glucose co-transporter, SGLT-1. This was verified by adding phloridzin, an inhibitor of SGLT-1, to the apical chamber to a final concentration of 0.5 mM. To determine if AMP rapidly upregulates glucose uptake by the epithelia, as we have previously reported in vivo and by intact small intestine (Kimura et al. 2005), a portion of the apical medium was replaced by a solution of 50 mM AMP in Ringers to achieve a final concentration of 20 mM. *Isc* was recorded and when stabilized, a portion of the apical solution was replaced by the 50 mM solution of glucose in Ringers for a final concentration of 20 mM. Blank inserts were exposed to identical changes in solutions and were used as controls; *Isc* remained stable in the control inserts.

Accumulation of ^14^C d-glucose was measured in triplicate or quadruplicate 2–12 h after enterocytes harvested from mice were added to inserts at densities of 4–5 × 10^6^ cells cm^−2^. For an uptake measurement, the culture medium was gently aspirated from the inserts and replaced with 37 °C Hank’s Buffered Salt Solution (HBSS) with 25 mM mannitol (control) or 25 mM adenosine. After 10 min the solutions were removed and replaced with an uptake solution consisting of the control solution with tracer concentration (4 μM) of ^14^C d-glucose. The cells were allowed to accumulate the labeled glucose for 4 or 5 min after which the uptake solution was removed, the epithelia were rinsed twice with cold HBSS-mannitol, lysed with 0.1 N sodium hydroxide and the lysates were transferred to scintillation vials. Scintillant (UltimaGold XR, PerkinElmer, Waltham, MA, USA or ScintiVerse II; Fisher Chemical, Pittsburgh, PA, USA) was added and accumulated radioactivity was measured as disintegrations per min by liquid scintillation counting.

The contributions of SGLT-1 and the facilitative glucose transporter GLUT2 to the adenosine induced increase in glucose accumulation were determined using 8 h epithelia prepared from mouse enterocytes. After the epithelia were exposed to 25 mM adenosine, glucose accumulation was measured in the presence and absence of 0.5 mM phloridzin or phloretin in the uptake solution to inhibit SGLT-1 and GLUT2, respectively. To determine if the increased accumulation of glucose involves trafficking of SGLT-1 from intracellular pools via a microtubule mediated mechanism (Khoursandi et al. 2004), nocodazole (10 μg/ml) was added to the 25 mM adenosine solution prior to measuring the accumulation of ^14^C d-glucose. The role of a cAMP mediated signaling pathway in triggering the increased glucose uptake was determined using epithelia prepared from mouse enterocytes that were exposed for 10 min to forskolin (100 μg/ml of the control solution) before measuring glucose accumulation.

Enterocytes were harvested from the jejunum of preterm pigs that were healthy and from pigs with necrotic lesions characteristic of NEC that were isolated to the colon. The harvested cells were cultured on inserts at 4–5 million cells per cm^2^. After 8 h the epithelia were used to measure ^14^C d-glucose accumulation with and without phloridzin (0.5 mM) included in the uptake solution.

### Statistics

Values are reported as mean ± SEM. An unpaired Student’s *t* test was used to compare glucose accumulation by control and adenosine or AMP stimulated epithelia. Data for accumulation of glucose by epithelia with and without exposure to adenosine and in the presence or absence of pharmacologic agents (phloridzin, phloretin, forskolin, and nocodazole) were analyzed by one-way ANOVA (Statistical Analysis System, Version 9.3; SAS Institute, Cary, NC, USA). When a significant treatment effect was detected, specific differences among treatments were identified by Duncan’s multiple range test. A value of *P* < 0.05 was considered as significant.

## Results

The harvesting, rinsing, enumerating, and adding of the cells to inserts requires 75–90 min after removal of the intestine. Importantly, only for the final 5–10 min of exposure to the dissociation/cell detachment solution did the cells become detached and solitary, which are conducive to anoikis. After this brief period the cells were kept cold before they were suspended in the DMEM, seeded onto inserts, placed in the incubator, and allowed to re-establish cell–cell adherence and form functional epithelia. The high viability of the cells indicates the short period of detachment and solitary existence and subsequent processing did not trigger anoikis.

There are numerous protocols for the harvest of enterocytes that use ethylenediaminetetraacetic acid (EDTA). Short-term (<1 h) exposure to 5 mM EDTA is not cytotoxic for Caco-2 cells (Quan et al. 1998). Although we harvested high numbers of enterocytes using 5 mM EDTA, the cells had low viability at 2 h, did not form epithelia, and accumulation of ^14^C d-glucose did not exceed background. Even concentrations of 1.5 mM EDTA resulted in low enterocyte survival at 2 h and diminished the abilities of the cells to accumulate glucose. HyQTase^®^ used to harvest the cells is reported by the supplier to contain 0.5 mM EDTA. The manufacture of the dissociation solution does not disclose the concentration of EDTA or sodium citrate.

The proximal 50 % of the small intestine of an adult mouse typically yields 10–20 million cells. Although the optimal ratio of the amount of tissue per volume of detachment solution was not determined, a volume of 10 ml of dissociation solution was considered adequate to harvest cells from an entire mouse small intestine. A similar ratio of dissociation solution relative to the amount of tissue was used to harvest cells from the segments of jejunum collected from preterm pigs.

### Microscopic characteristics

**Fig. 1:**
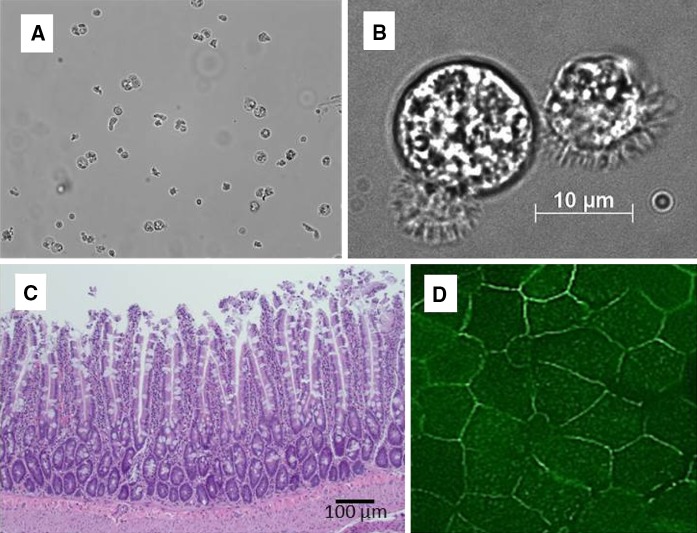
Light micrographs of enterocytes harvested from the small intestine of a C57/BL mouse (**A** ×200) and an individual differentiated enterocyte with microvilli (**B** ×1,000), and hematoxylin and eosin stained section from the jejunum of a mouse after the harvest of enterocytes showing that after the harvest the epithelium is removed from the upper villus region (**C** ×100). Establishment of cell–cell connections and reorganization of the harvested cells into an epithelium 6 h after seeding inserts is evident by immunofluorescent staining of ZO-1 in the membrane domain (**D**)

**Fig. 2:**
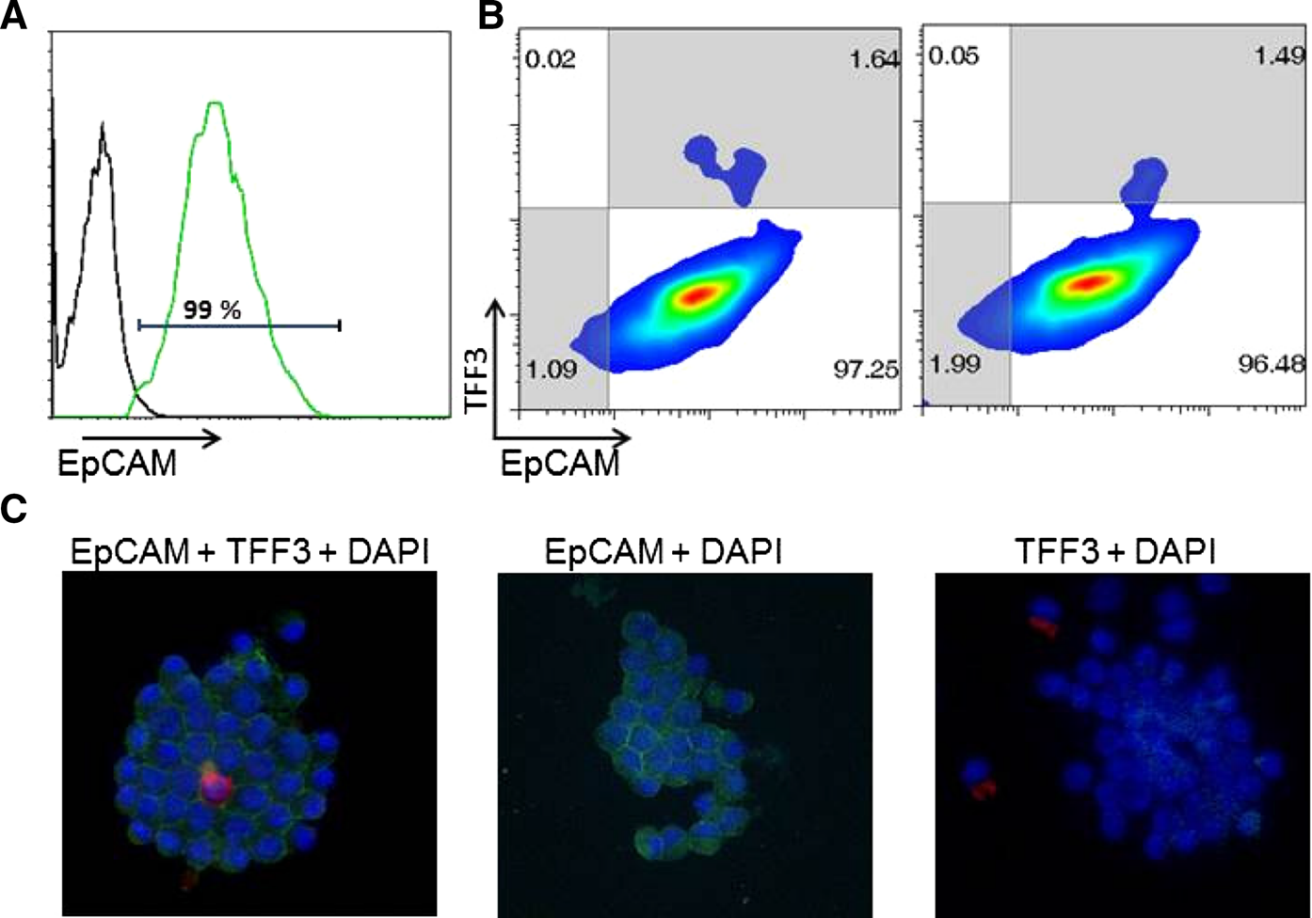
Freshly harvested mouse enterocytes were analyzed by flow cytometry (**A**, **B**) and immunocytochemistry **C** to determine the percentage of cells that expressed EpCAM as a marker of epithelial origin and TFF3 to identify goblet cells among the harvested cells. **A** Representative FACS histogram of mean fluorescence intensity for EpCAM expression indicated by green histogram. The *black* histogram represents the isotype control. **B** Representative FACS plot for EpCAM (*x axis*) vs TFF3 (*y axis*) expression (*left panel*). *Right panel* shows EpCAM expression vs TFF3 isotype control. **C** Immunocytochemical analysis of harvested epithelial cells demonstrating EpCAM (*green*), TFF3 expression (*red*) and cell nuclei stained using DAPI (*blue*) (*left panel*). *Middle panel* shows TFF3 isotype control in the presence of EpCAM staining. *Right panel* shows EpCAM isotype control in the presence of TFF3 staining

The majority of harvested cells were solitary (>90 % in most preparations), with only occasional groupings of 2 or more cells in the suspensions used to prepare epithelia (Fig. 1A). The suspensions appeared to include a small percentage (<1 %) of other cell types (e.g., goblet cells), which is consistent with the diversity of cells in the upper villus epithelium. We demonstrated using flow cytometry and EpCAM, which is a marker for cells of epithelial origin, that 98.4 % of the harvested cells were positive for EpCAM expression (Fig. 2A). To determine what fraction of these cells might be goblet cell we assessed TFF3 expression and learned less than 1 % of the harvested cell were positive for TFF3 (Fig. 2B, right panel). This expression was also confirmed by immunocytochemistry using anti-EpCAM and TFF3 antibodies (Fig. 2C, left panel). The harvested cells retained microvilli that were restricted to an isolated region of the cell membrane (Fig. 1B). Histological evaluation of mouse small intestine segments after exposure to the dissociation/cell detachment solutions indicated that the majority of the cells originated from the upper 20–40 % of the villus (Fig. 1C). Viability of the harvested enterocytes immediately prior to plating assessed by trypan blue exclusion was routinely >90 %, with most preparations having >95 % viable cells. Viability did not differ among cells harvested from the mice and the preterm pigs.

The cells settled rapidly in the inserts and began to form epithelia within 30 min, with a cohesive layer evident within 2 h. Importantly, the enterocytes retained a distinct brush border membrane and reformed tight junctions as evident from the accumulation of ZO-1 at the periphery (Fig. 1D). The majority of cells were viable 12 h after plating on the inserts. Thereafter, viability declined and the epithelia lost structural integrity, which was evident by detachment from the insert, creating denuded areas.

### Functional characteristics of epithelia prepared from mouse enterocytes

**Fig. 3:**
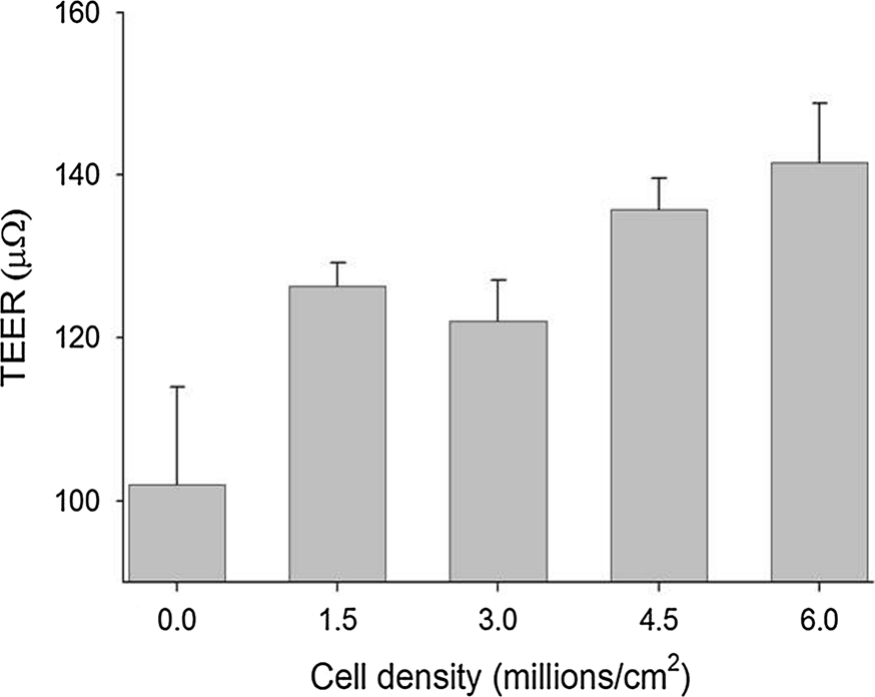
Transepithelial electrical resistance (TEER) measured using an EVOM^®^ Epithelial Voltohmmeter 12 h after preparing epithelia using enterocytes harvested from the jejunum of a pig. TEER at densities of 1.5 million and higher were significantly (*P* < 0.05) higher than control inserts without cells

TEER was significantly higher at 2 h after adding mouse enterocytes to inserts compared with control inserts without enterocytes. Seeding cells at densities exceeding 5 × 10^6^ cells per cm^2^ did not cause appreciable further increases in TEER (Fig. 3).

**Fig. 4:**
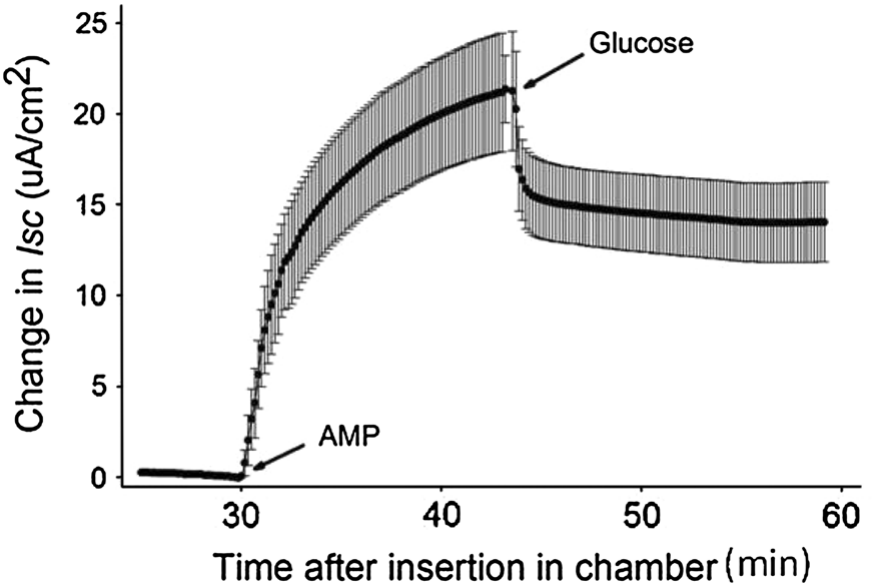
Changes in short circuit current (*Isc*) after addition of adenosine monophosphate (AMP) and glucose to four epithelia prepared from enterocytes harvested from an adult C57BL/B6 mouse and after 7 h of culture. Values are mean ± SEM for the difference between I*sc* at each time point minus the average value for the 5 min prior to the addition of AMP

Exposure of epithelia prepared using mouse enterocytes to AMP elicited a rapid and dramatic change in *Isc* (Fig. 4). The *Isc* response to AMP could also be elicited by forskolin (data not presented). The subsequent addition of glucose to the apical chamber elicited an opposing change in *Isc* that could be inhibited by the presence of phloridzin.

**Fig. 5:**
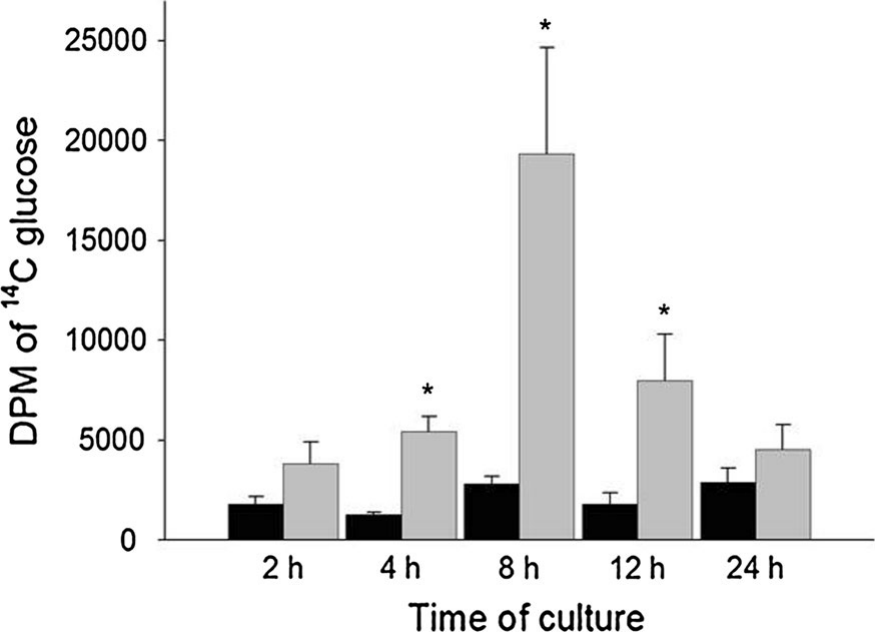
Accumulation of ^14^C d-glucose by epithelia prepared using mouse enterocytes that were cultured on inserts for 2–24 h before exposure to 25 mM concentrations of mannitol (control *black bars* n = 3) or adenosine (*gray bars* n = 3) for 10 min before measuring accumulation. *Asterisks* indicate that a difference (*P* < 0.05) was detected between control and adenosine-exposed epithelia

Accumulation of ^14^C d-glucose by epithelia prepared from mouse enterocytes exposed to mannitol (unstimulated) did not vary significantly when measured at different times during the first 24 h after plating on the inserts (Fig. 5). In contrast, the increase in d-glucose accumulation in response to 25 mM adenosine was maximal at 8 h, although a significant increase relative to control epithelia was evident after only 4 h of culture. The decline in the response to adenosine at 12 h suggests the useable lifespan of epithelia prepared from mouse enterocytes is limited to <12 h.

**Fig. 6:**
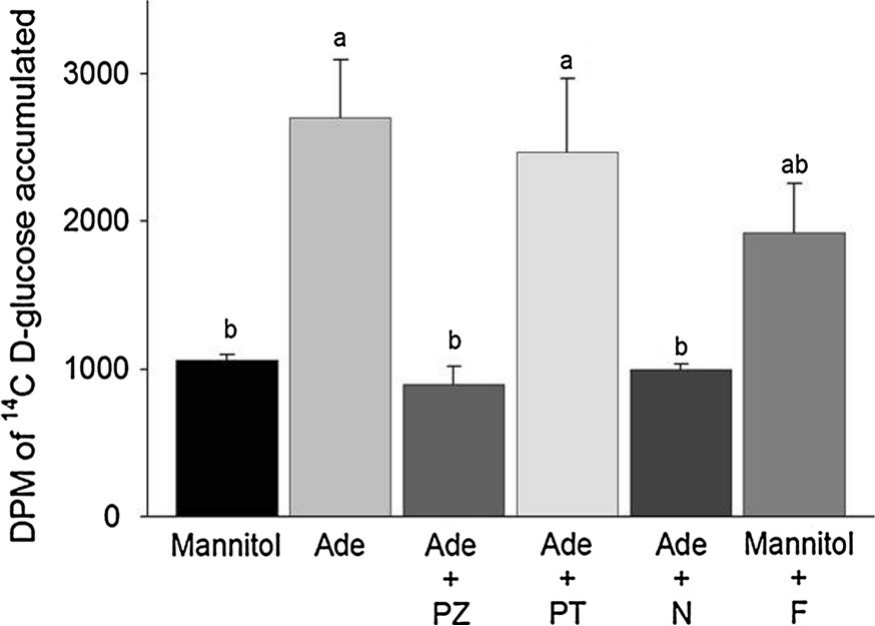
Accumulation of ^14^C d-glucose by epithelia prepared using mouse enterocytes and pre-exposed for 10 min to 25 mM mannitol (control n = 4) or adenosine (Ade n = 4) with and without pharmacological agents (n = 4) to inhibit SGLT-1 [phloridzin (0.5 mM), PZ] and the facilitative glucose transporter, GLUT2 [phloretin (0.5 mM), PT], to disrupt microtubules and thereby inhibit vesicle trafficking [nocodazole (10 μg/ml), N]. The role of intracellular production of cAMP in increasing glucose accumulation was examined using forskolin (100 μM; Mannitol + F). *Bars* with *different letters* are significantly different (*P* < 0.05) based on Duncan’s test

The increased accumulation of glucose by epithelia prepared from mouse enterocytes in response to adenosine was inhibited by phloridzin, but not phloretin (Fig. 6) implicating SGLT-1, but not GLUT2 as the responsive glucose transporter. Glucose accumulation was also stimulated by exposure to forskolin. Moreover, nocodazole inhibited the response of epithelia to adenosine, as we reported for intact tissues (Kimura et al. 2005). Collectively, these findings indicate the increase in glucose accumulation stimulated by adenosine involves a signaling pathway that includes cAMP production and may require trafficking of SGLT-1 from intracellular pools to the apical membrane via microtubules.

### Functional attributes of epithelia prepared from preterm pig enterocytes

**Fig. 7:**
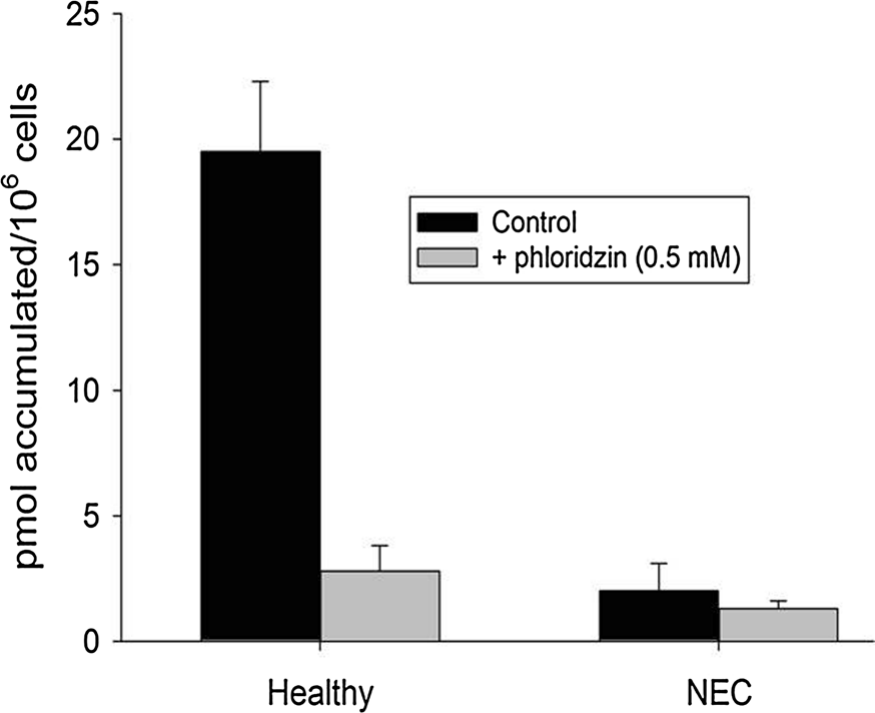
Accumulation of ^14^C d-glucose in the presence and absence of phloridzin (0.5 mM) to inhibit SGLT-1 by 8 h epithelia that were prepared using enterocytes harvested from the jejunum of pre-term pigs that were either healthy or had necrotizing enterocolitis (NEC) that was restricted to the colon

Epithelia prepared using enterocytes from healthy preterm pigs also exhibited responses to AMP, forskolin, and glucose that were similar to those of epithelia prepared from mouse enterocytes (data not presented). Accumulation of d-glucose by epithelia prepared using enterocytes harvested from the jejunum of healthy preterm pigs was more than seven-fold higher in an unstimulated state (no exposure to adenosine or AMP) compared with epithelia prepared from pigs in the same litter, but with NEC detected in the colon (Fig. 7). The higher unstimulated glucose accumulation by epithelia from healthy pigs was inhibited by phloridzin, suggesting enterocytes of healthy pigs have more functional SGLT-1 in the apical membrane.

## Discussion

The characteristics of the upper villus enterocytes are the result of interactions between genetic determinants and a combination of signals originating from the complex milieu of the intestinal lumen and from the host. Epithelia prepared using harvested differentiated enterocytes that are plated at confluent densities provide an alternative approach for comparative studies of nutrient transport by the upper villus epithelium. Although studies using the epithelia are limited to <12 h after plating, this is sufficiently long to explore both genomic and non-genomic responses of the villus epithelium to luminal and systemic stimuli and exceeds the lifespan of intact tissues. Preparation of the epithelia is simple, does not require stringent sterility, specialized equipment, or complex and multiple steps for isolation of enterocytes, and produces functional epithelia within 6–8 h after harvest that are more likely to represent the characteristics of the in situ villus epithelium than existing cell lines. Although the harvested cells are not pure enterocytes, the <2 % that are not enterocytes are mostly goblet cells, which is similar to the distribution of cells in the upper villus epithelium. It is possible that the approach can be adapted to harvest enterocytes from lower regions of the villus. Although not determined, the resulting epithelia from such cells may have different characteristics than those prepared using enterocytes from the upper villus. Importantly, the approach can be replicated as indicated by our performing studies in laboratories located in three different sites using different species and health states, as well as vendors of culture media and supplies.

The inhibition of ^14^C d-glucose accumulation and glucose-induced *Isc* by epithelia exposed to phloridzin, but not to phloretin, demonstrates that glucose uptake by upper villus enterocytes is mediated by SGLT-1, not by GLUT2. The rapid increase in glucose uptake stimulated by exposure to adenosine and AMP was inhibited by nocodazole, which corroborates our findings with intact tissues (Kimura et al. 2005) and reports that microtubules play a role in the upregulation of SGLT-1 via trafficking from intracellular stores (Khoursandi et al. 2004; Suzuki et al. 2006). Furthermore, the responses of the epithelia prepared from harvested enterocytes to adenosine and AMP coincides with the responses we have reported for intact tissues (Kimura et al. 2005), but contrast sharply with the >70 % reduction in glucose accumulation when Caco-2 cells are exposed to adenosine (our unpublished data). Adenosine stimulates glucose uptake by IEC-6 cells (our unpublished data). However, IEC must be cultured aseptically for 12–14 days, with dexamethasone added to the culture medium for the final 24 h to induce cell differentiation and expression of SGLT-1, and for the cells to develop the response to adenosine. The change in *Isc* by the epithelia in response to adenosine is a novel finding and as yet is unexplained.

An early concern was whether the cells would form epithelia with a consistent orientation of the cells. The data from the Ussing chamber studies and particularly the induced changes in *Isc* indicate the harvested enterocytes do establish polarized epithelia with distinct apical and basolateral domains, similar to cultured cell lines. The low TEER for epithelia prepared from harvested enterocytes is indicative of a leaky epithelium and is similar to values for small intestine epithelia (Mineo et al. 2002), for monolayers of IEC-18 (Konsoula and Barile 2007), and for primary cultures of enterocytes (Weng et al. 2005). The low TEER is in contrast with the high values measured for monolayers of transformed cell lines commonly used as models for enterocytes, such as Caco-2 (Fuller et al. 2007), HT-29 (Maher and McClean 2006), and T84 (Kasuiga et al. 1998) cells. Despite the low TEER for epithelia prepared from harvested enterocytes, it was possible to detect significant and consistent changes in *Isc* in response to AMP, adenosine, glucose, and forskolin. Moreover, the responses are consistent with our report of non-genomic increases in glucose accumulation by intact tissues (Kimura et al. 2005), but of greater magnitude, hence sensitivity.

The more than seven-fold higher glucose accumulation by epithelia prepared using enterocytes from healthy preterm pigs compared with epithelia prepared using cells harvested from littermates with necrotizing enterocolitis exceeds the twofold difference we measured using intact tissues from pigs with and without comparable severities of NEC (Buddington et al. 2008). Moreover, the responses of epithelia prepared from mice to adenosine and forskolin exceeded those we measured using intact mouse tissues (Kimura et al. 2005). These findings indicate that epithelia prepared using harvested enterocytes provide a more sensitive approach than intact tissues for evaluating the carrier-mediated absorptive functions of the enterocytes lining the upper villus, the impact of disease state, and the responses to luminal conditions and host regulatory molecules.

The inability of newly (<2 h) plated enterocytes to increase glucose accumulation in response to adenosine or AMP may be related to the disruption of the cell–cell and cell–matrix interactions caused by the isolation of enterocytes, which would also disrupt the cytoskeleton (Schreider et al. 2002) and the ability to traffic SGLT-1 to the apical membrane. The gradual increases in TEER and the responses to adenosine and AMP provide evidence for the establishment of the cell–cell contacts associated with an epithelium. Although not verified, this process would be initiated by the establishment of adherens junctions via E-cadherin (Schreider et al. 2002) and re-organization of the cytoskeleton (Mège et al. 2006). The evidence for an epithelium within 2 h after plating the harvested enterocytes is consistent with the rapid formation of epithelia by MDCK cells plated at a density sufficient for confluence (Suzuki et al. 2006).

Cell to cell contact is essential for survival of epithelial cells (Hofmann et al. 2007). Anoikis is triggered in 20–30 % of intestinal epithelial cells within 30 min after detachment from the basement membrane and the loss of cell–cell adherence, with the majority of cells dead by 3 h (Fouquet et al. 2004). The increased viability for the enterocytes used to prepare epithelia suggests that the short period needed to harvest and plate the cells, the low concentration of EDTA used, and other as yet unknown factors inhibited anoikis and allowed the cells to rapidly re-establish cell–cell junctions after plating.

A decrease in epithelial integrity after 12 h of culture was obvious by the appearance of denuded areas of the epithelium and by reductions in glucose induced currents and accumulation. These findings indicate the lifespan of the epithelia is limited, which is consistent with reports for the short term viability of enterocytes harvested from the dog (Weng et al. 2005) and polarized duodenocytes from rats and human biopsies (Chew et al. 1998). Even if the lifespan of the enterocytes, hence the resulting epithelium, could be increased, it is likely that the cells would adapt to culture conditions and would have characteristics that no longer are representative of the in situ environment.

Advances in culture media and supplements and alternative approaches for the harvest and culture of enterocytes from other vertebrates are expected. These will include identifying specific nutrients and growth and attachment factors that will accelerate and strengthen the cell–cell and cell–matrix adherence that develop as the isolated enterocytes form epithelia.

## Abbreviations

AMP: Adenosine 5′-monophosphate disodium
DAPI: 4′,6′-Diamidino-2-phenylindole
DMEM: Dulbecco’s modified Eagle’s medium
DPBS: Dulbecco’s phosphate buffered saline
EpCAM: Epithelial cell adhesion molecule
EDTA: Ethylenediaminetetraacetic acid
Fc: Fc immunoglobulin receptor
FACS: Flow cytometry
FITC: Fluorescein isothiocyanate
HBSS: Hank’s balanced salt solution
IgG2: Immunoglobulin G2
IEC: Intestinal epithelial cell
HEPES: 4-(2-Hydroxyethyl)-1-piperazineethanesulfonic acid
*Isc*: Short circuit current
PBS: Phosphate buffered saline
PE: Phycoerythrin
SGLT-1: Sodium-glucose cotransporter 1
TEER: Transepithelial electrical resistance
TFF3: Trefoil factor 3
ZO-1: Zonula occludens**-**1
